# Using MD Simulations to Understand the Impact of Directed Evolution on Oxygen Affinity in Amine Oxidases

**DOI:** 10.1002/open.70164

**Published:** 2026-03-29

**Authors:** Christopher R. Field, Rowan Lindeque, Jonathan P. Dolan, Julie Østerby Madsen, Sam Hay, Nicholas J. Turner, John M. Woodley, Sebastian C. Cosgrove

**Affiliations:** ^1^ Future Biomanufacturing Research Hub Manchester Institute of Biotechnology Department of Chemistry School of Natural Sciences University of Manchester Manchester UK; ^2^ Department of Chemical and Biochemical Engineering Technical University of Denmark Lyngby Denmark; ^3^ School of Chemical and Physical Sciences Keele University Keele, Staffordshire UK; ^4^ Manchester Institute of Biotechnology Department of Chemistry University of Manchester School of Natural Sciences Manchester UK

## Abstract

Mutants of the amine oxidase from *Aspergillus niger* were tested to ascertain the need for molecular oxygen across the family of mutant enzymes. Two of the variants were selected, namely D5 and D11, which had 5 and 10 mutations, respectively, versus the wild type. Experimental observations showed a difference in oxygen requirements between variants. Molecular dynamics simulations were then used to give a potential explanation for this difference in observed activity at a molecular level.

## Graphical abstract

A combination of experimental and computational experiments has been used to understand oxygen needs for evolved amine oxidase enzymes. Two variants of amine oxidase from *Aspergillus niger* were shown to have different oxygen requirements in the oxidation of a model substrate, with molecular dynamics simulations used to model the differences between the two variants.

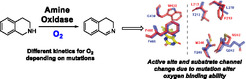

## 1 Introduction

Oxidation is a fundamental chemical reaction [1]. In industry, oxidation processes provide intermediates that can be used in synthetic routes toward more complex chemicals with a range of uses. Despite this, chemists often avoid synthetic routes which contain oxidative steps due to the problems associated with available oxidation techniques [2]. Historically, oxidation chemistry has relied heavily on stoichiometric transformations dependent on transition metals, such as chromium and manganese, hypervalent iodine (e.g., Dess‐Martin periodinane) and Swern‐type oxidations. These frequently operate at high temperatures, proceed with low chemoselectivity, and produce stoichiometric amounts of waste. Catalytic oxidation offers more efficient ways to perform these reactions, but the use of precious metals has numerous issues associated with it, not least increasing scarcity, as well as toxicity [3].

Taking inspiration from nature has enabled the development of oxidative biocatalysis, whereby isolated enzymes perform oxidation reactions with the efficiency of natural systems but under biological conditions [4]. The aim now is to get them to convert effectively under process conditions. One area that has seen significant development is the oxidation of amines to imines using monoamine oxidase (AmOx) enzymes [5, 6]. The synthetic value of imines for the synthesis of chiral amines, one of the most important moieties in synthetic chemistry [7], underlines the importance of developing new AmOx biocatalysts. Therefore, developing a better understanding of how these enzymes perform under process conditions is essential to allow their potential to be realized.

AmOx enzymes broadly fit into two classes: copper radical AmOx, which are dependent on Cu(II), and Flavin‐dependent AmOx, which usually have a tightly bound flavin in the active site [8]. Both are dependent on molecular oxygen as a cosubstrate, further highlighting their synthetic appeal by avoiding the need for stoichiometric, exogenous oxidants. The need for oxygen, however, can limit synthetic performance, since enzymes naturally work in water. While the maximum aqueous concentration of oxygen under ambient conditions is approximately 270 μM (~8 mg L^−1^) at 25°C [9], numerous oxidases have been shown to require significantly higher concentrations to reach their kinetic optima [10].

One way to understand this and quantify it in relatively simple terms is to compare the Michaelis constant for oxygen (*K*_MO_). Previous work from several groups, including the Woodley group, have used a variety of techniques to determine *K*_MO_, and in many cases, it has been shown to be significantly higher than the available oxygen under ambient conditions [11]. For example, a recent review presented the *K*_MO_ values for 10 oxygen‐dependent enzymes, ranging from 0.25 to 3 mM [10], which included an AmOx with a reported *K*_MO_ of 0.33 mM [12]. One thing yet to be studied in the context of flavin‐dependent AmOx is whether laboratory directed evolution for improved activity toward synthetic substrates impacts on the ability to bind and react with oxygen, although it was demonstrated within galactose oxidase variants by Birmingham et al. previously [13]. Stull recently demonstrated through ancestral sequence reconstruction that an amine oxidase active toward a nicotine derivative naturally evolved improved oxygen binding from a parent dehydrogenase, and that retro‐fitting dehydrogenase ancestors with “oxidase” residues could impart oxidase activity [14]. Interestingly, an ancestral mutant with 21 mutations was converted from a dehydrogenase to an oxidase, showcasing the impact that select mutations can have on oxygen binding. This supports other work from the Stull group whereby flavin‐dependent dehydrogenases which use cytochrome‐c electron acceptors have been evolved to use oxygen only as the terminal oxidant [15, 16].

To ascertain whether this is a general trend among oxidases, and whether laboratory evolution has a similar impact, two variants of monoamine oxidase from *Aspergillus niger* (MAO‐N) have been studied. The MAO‐N mutants were engineered from the same wild type enzyme for increased substrate tolerance towards non‐natural amines, which produced a panel of variants [17]. This study focusses on the D5 and the D11 variants. The D5 variant had five mutations compared to the wild‐type enzyme (I246M/N336S/M348K/T384N/D385S) [18] and showed oxidative activity toward cyclic amines for the first time. Further engineering afforded the D11 variant, which had an additional four mutations and an alteration of the methionine at the D5 246 position (F210L/L213T/M242Q/I246T/W430G) [17]. These mutations expanded the active site cavity volume from 102 Å^3^ in D5 to 161 Å^3^ in D11. The end result meant that D11 could accept a variety of much larger organic amines as substrates [19]. Herein, we explore the effect these mutations may have had on the oxygen requirements of these variants.

## 2 Results and Discussion

**FIGURE 1:**
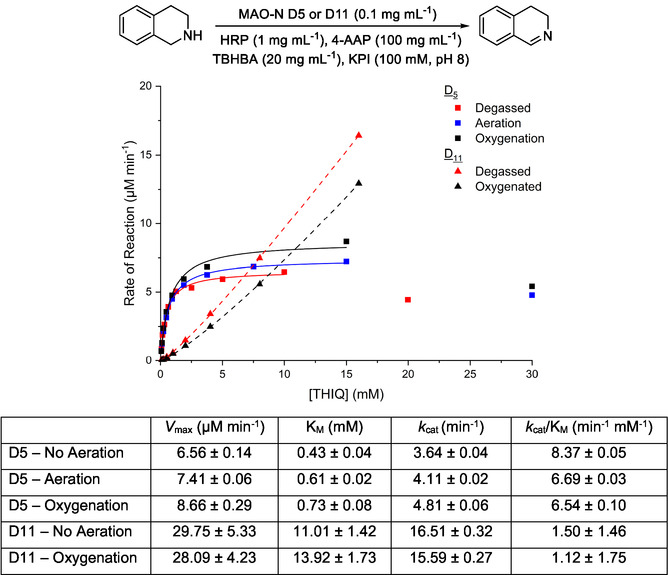
Effect of oxygenation of buffer on apparent *K*_M_ for THIQ with MAO‐N variants D5 and D11. Full experimental details can be found in the Materials and Methods. 4‐AA*P* = 4‐aminoantipyrine; TBHBA = 2,4,6‐tribromo‐3‐hydroxybenzoic acid.

To compare D5 and D11 as closely as possible, a common substrate was chosen in tetrahydroisoquinoline (THIQ). This cyclic amine is not chiral, so oxidation would yield a single product, simplifying analysis. Apparent kinetics were determined for THIQ with each variant using a standard horseradish peroxidase (HRP) assay for H_2_O_2_ detection, which is the byproduct from enzymatic turnover [20]. The buffer used was either stirred without sparging, stirred and sparged with air, or else stirred and sparged with oxygen, for 10 min prior to sampling, and continuously thereafter to maintain saturation. Three sets of data were then recorded: kinetics with no aeration, with air saturated buffer, and with oxygen saturated buffer (Figure 1). It became clear that the level of aeration within the buffer used to record the measurements impacted on the apparent kinetics. Kinetics for the D5 variant clearly showed that increasing oxygen supply to the buffer resulted in an increase of both apparent *V*_max_ and apparent *K*_M_. Interestingly, the observed *k*_cat_/*K*_M_ (= *k*_cat,app_/*K*_M,app_) values altered slightly across conditions for each enzyme and experiment, suggesting perhaps a change in binding order during oxygen binding. Indeed, Trimmer et al. made similar observations in the study of an engineered amino acid oxidase [21], while Adachi et al. suggested that flavin could interact through more than one pathway with oxygen during spermine oxidation by human spermine oxidase based on their kinetic analysis [22]. It should be noted that at concentrations above 16 mM, THIQ resulted in suspected inhibition of HRP used in the assay. This was observed for both the D5 and D11 variants. As a result of the inhibition, only the start of the curve could be captured for the D11 variant. The fitting of the data (both *R* ^2^ = 0.99) for the D11 variant suggests, as expected, that enhanced oxygenation of the buffer results in an increase in *K*_M_ compared to D5. However, there are limitations in the HRP assay due to the high affinity that was observed. Nonetheless, the substantial difference in apparent values for each variant illustrates that the introduction of the five additional mutations in the D11 variant has an impact on oxygen binding.

**FIGURE 2:**
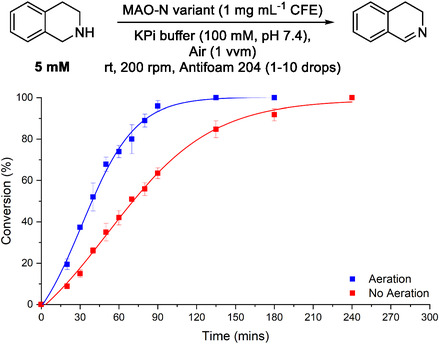
Non‐aerated vs. aerated MAO‐N D11 oxidation of THIQ in Applikon reactor. Reaction conditions are detailed in Materials and Methods.

This significant difference in values between the two MAO‐N variants (D5 vs. D11 values) demonstrates that they are oxygen‐limited. Under ambient conditions, the dissolved oxygen concentration is limited which will impact the effectiveness of the enzymes, and this is exacerbated through depletion of the available oxygen over time. To address this, the reaction was run in a reactor with continuous gas sparging (150 mL scale) to understand how aeration would affect conversion over time, with the reactor left open to air to maintain constant pressure. While improved oxygen supply under these conditions would not increase the maximum concentration of oxygen at atmospheric pressure, continuous depletion over time would be mitigated slightly, allowing the enzyme to maintain faster rates. To control oxygen supply, an Applikon reactor was used as it could simultaneously monitor and maintain dissolved oxygen concentration throughout the reaction [11]. As can be seen for the D11 variant in Figure 2, simple agitation of the reaction with no additional oxygen supply took considerably longer to achieve full conversion (red data points) than if the reaction that had a continuous oxygen supply via the in‐built sparging device (blue data points). Aeration allowed full conversion to the imine to be reached in approximately 90 min, whereas agitation with the impeller had only reached 62% at the same point in time. Sparging with 100% oxygen was attempted but our results were highly inconsistent and could not be repeated from one run to the next. During each run, a yellow gum quickly formed and stuck to the outside of the reactor vessel. We speculate that this may have been either starting material or product, leading to the inconsistent results observed during the 100% oxygen reactions. While we did not confirm the nature of the gum, this demonstrated that using the pure oxygen gas impacted reaction outcome, so we did not pursue this further.

**FIGURE 3:**
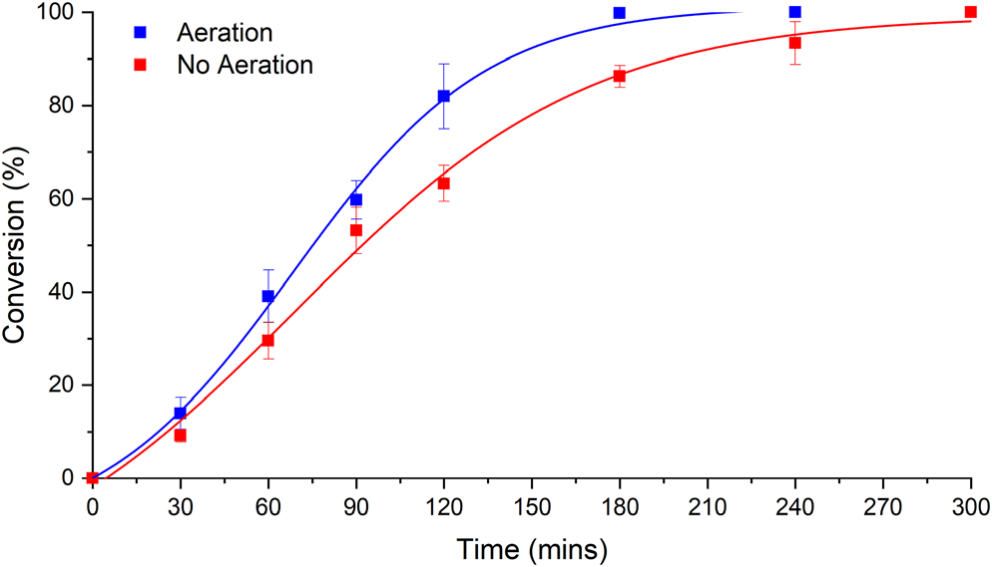
Non‐aerated vs. aerated MAO‐N D5 oxidation of THIQ in Applikon reactor. Reaction conditions are detailed in Materials and Methods.

Comparison of these results with MAO‐N D5 under identical conditions showed little difference (Figure 3). While aeration did increase the rate of reaction (blue data points), there was a much smaller effect on reaction rate versus the experiment with no aeration (red data points). For example, 100% conversion was reached after 180 min in the aerated reaction, but just using agitation alone reached 86% in the same timeframe.

These two sets of experiments implied that improved oxygen supply impacts the performance of the enzymes. However, depending on which AmOx variant was used, this difference was more pronounced, matching the results of our observed kinetic reactions.

**FIGURE 4:**
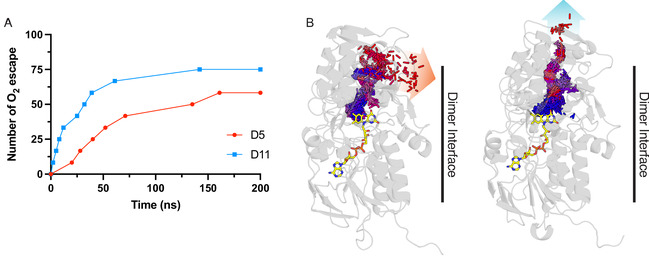
(A) Cumulative oxygen escape events with respect to time. (B) Movement of oxygen from the center of a MAO‐N monomer binding site to the solvent surrounding the protein. Oxygen trajectories are shown as a spectrum from blue (0 ns) to red (time point at which oxygen escapes). Reduced FAD is shown in yellow without hydrogen atoms for visual clarity. The paths taken by oxygen include the primary and secondary substrates tunnel (orange and blue, respectively).

To further explore the possible underlying molecular reasons for the observed experimental results, molecular dynamic simulations were used to understand the behavior of oxygen in the context of the two MAO‐N variants. Molecular oxygen was positioned within the active sites of MAO‐N variant dimers, tracking the path traveled within the protein and the time taken for the oxygen to “escape” into the surrounding solvent. A total of 6 replicates were run for each variant (3 × 200 ns; 3 × 100 ns; 2 oxygen molecules per simulation). The oxygen was considered to have escaped when the magnitude of the vector between the binding site and the oxygen was consistently greater than the average length of the given escape tunnel by at least 2 ns. In the case of D11, 9 oxygen molecules escaped with an average escape time of 35.5 ns, whereas for D5 only 7 escaped, and the average time was more than double (72.1 ns) (Figure 4a). The majority of the escapes occurred through the primary substrate channels, as identified by Curado‐Carballada et al. (Figure 4b) [23, 24].

**FIGURE 5:**
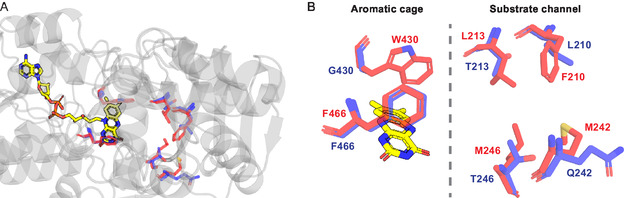
Overlapped residues between the MAO‐N D5 and D11 variants. (A) Relative locations in the protein structure. (B) Specific proximity in the aromatic cage and substrate channel. D5 residues red, D11 blue, and FAD yellow.

Significantly, in the D11 variant, the oxygen always escaped via a tunnel which contained three mutations which were not present within the D5 variant (F210L, L213T, M242Q). Another substrate channel residue (I246M in D5; I246T in D11) interacted with each oxygen that escaped for both variants (Figure 5A). Interestingly, the W430G residue only present in D11 was not seen to interact with oxygen during any of the simulations, whereas W430 did interact with oxygen in every D5 simulation. This tryptophan residue forms an aromatic cage with a proximal phenylalanine (F466) in D5, with both sat perpendicular to the isoalloxazine moiety of the FAD in the active site (Figure 5B). Overall, these results indicate D11 variant‐exclusive mutations F210L, L213T, M242Q, I246T (substrate channel), and W430G (binding site aromatic cage) as possible contributors towards the experimentally observed drop in oxygen affinity.

In comparison with other amine oxidases (i.e., copper‐dependent amine oxidase), there are two distinct but favored pathways through which flavin AmOx proceeds through, hydride abstraction or single electron pathways (also nucleophilic pathways are proposed) [8]. In both the proposed radical and hydride pathways, only the oxygen directly bonds to the flavin, underlining the importance of the modeling undertaken here. While substrate interactions are clearly important, the work here proposes that flavin–oxygen interactions are just as important when considering evolution strategies. The modeling here also helps to offer some postrationalization of the observed experimental results reported above.

The collective data disclosed above begins to paint a picture of the importance of oxygen during oxygen‐dependent enzyme evolution. Mechanistically, it is a substrate for the reaction and as such should be thought of in this way. The difference obviously is that the supply of increased amounts of gaseous substrates is considerably more challenging than organic molecules. The W430G mutation was input to significantly expand the active site cavity during the evolution of the D11 variant, permitting larger organic molecules to enter the active site. Our modeling data suggests the tryptophan in the earlier mutants is important for oxygen‐binding, so this specific glycine mutation has a severe impact here. Specific residues that are important for oxygen‐binding in oxidases have been discussed previously [25]. On a mechanistic level, the rate‐limiting step of flavin oxidation is reported to be the orientation of oxygen with the reduced flavin N(5)‐C4a bond. An increased positive charge surrounding this area is thought to be important to enable this orientation and initial reaction between flavin and molecular oxygen [26]. A sarcosine oxidase activity was improved by 250‐fold through a lysine to asparagine mutation proximal to the flavin [27, 28]. Also, in agreement with the molecular dynamics observations discussed here, mutation of a glutamate to glycine widened the access tunnel for oxygen in a putrescene oxidase, and in doing so, the observed *K*_MO_ increased from 0.246 to 0.296 mM [29]. These observations help to further contextualize our experimental observations and highlight the importance of considering oxygen during enzyme evolution. These are important observations with regard to the improvement of oxidase enzymes via directed evolution, in that oxygen plays an integral role as a substrate and must be considered when evolving toward organic molecules for synthesis.

## 3 Conclusion

In summary, we have shown that two mutants derived from the wild‐type monoamine oxidase from *Aspergillus niger* (MAO‐N) have different oxygen needs during catalysis. The D5 mutant has five mutations versus the wild type and is impacted less by improved oxygen supply than the D11 mutant, which works more effectively when oxygen supply is increased during reaction. This can also be observed via the apparent kinetic measurements, with different values obtained for the same substrate depending on the oxygen saturation of the media. The observed values point toward the possibility of alternative mechanistic pathways occurring to the established ping‐pong mechanism for some oxidases, with apparent sequential kinetics [21, 22], but this requires further experimental validation. Simulations have started to help ascertain which residues may be important for binding within the MAO‐N active sites, with aromatic residues positioned around the flavin cofactor seemingly key. Further work is ongoing to determine more accurate kinetic measurements for oxygen with the MAO‐N variants, while also trying to understand further the underlying mechanism by which oxygen is bound in amine oxidases.

## 4 Materials and Methods

Unless otherwise stated, all chemicals were purchased from commercial suppliers (Acros UK, Alfa Aesar, Fisher UK, Fluorochem, and Sigma–Aldrich) and used as received. The MAO‐N variants were expressed and grown as per previous studies [30]. Plate assays were performed using TECAN Infinite 200 Pro M Nano. Gas chromatography analysis was undertaken on an Agilent 7820A GC‐FID system. The column used was an Agilent HP‐5 (30 m × 0.32 cm × 0.25 μm).

### 4.1 Kinetic Measurements

The specific activity, kinetics constants, and conversion rates of the MAO‐N variants were measured using a 4‐AAP‐TBHBA‐HRP coupled assay. A 2‐fold serial dilution of THIQ from 128 mM was prepared using KPi buffer (100 mM, pH 8) containing HRP (1 mg mL^−1^), 4‐aminoantipyrine (4‐AAP, 100 mg mL^−1^), and 2,4,6‐tribromo‐3‐hydroxybenzoic acid (TBHBA, 20 mg mL^−1^). 90 µL of each dilution was added to a 96‐well plate followed by MAO‐N (10 µL of 1 mg mL^−1^, final conc: 0.1 mg mL^−1^). The production of 4‐AAP/TBHBA dye (*ε* = 29 400 L mol^−1^ cm^−1^) was monitored at 510 nm using TECAN Infinite 200 Pro M Nano plate reader at 37°C in triplicate. For the kinetic characterization of the proteins, the kinetic constants *V*_max_ and *K*_M_ were determined by fitting the data to the Michaelis–Menten equation using OriginPro 2019b software.

### 4.2 MAO‐N Tetrahydroisoquinoline Oxidation Reactions

Reactions were carried out in a 150‐mL (liquid volume) my‐Control stirred tank reactor (Applikon Biotechnology B.V., Netherlands), as illustrated here [11]. An Ismatec Reglo Independent Channel Control peristaltic pump (Cole‐Parmer, USA) was used to supply the reactor with an enzyme feed (1 g L^−1^ AmOx CFE, 100 mM pH 7.4 potassium phosphate buffer). The reactor was sparged at 1 vvm (volume gas per volume reaction liquid per minute) with gas (a 4:1 mixture of nitrogen and oxygen to represent atmospheric air composition) and was agitated at 1000 rpm to ensure well‐mixed conditions. Foam formation in the reactor was controlled by manual dropwise addition of Antifoam 204. Oxygen saturation (%) in the reactor was monitored and logged using a robust optical oxygen probe (Pyroscience AT GmbH, Germany). The probe was calibrated by saturating the reaction media, prior to initiation of the reaction by addition of the enzyme, with nitrogen to achieve 0% oxygen saturation and, separately, pure oxygen to achieve 100% oxygen saturation. Percentage oxygen saturation was converted to dissolved oxygen concentration (mM) using the Henry's Law constant of oxygen in water (1.2 × 10^−5^ mol m^−3^ Pa^−1^). During operation, samples (490 μL) were taken from the reactor at regular intervals, using 5 M NaOH (10 μL) to quench the reactions and then EtOAc (500 μL) to extract the organics. The samples were analyzed by GC‐FID.

### 4.3 Molecular Dynamics Simulations

Full details of the simulations can be found within the supporting information.

## Supporting Information

Additional supporting information can be found online in the Supporting Information section. **Supporting Fig. S1**: *ColabFold* structures of the complete sequences for the A) D5 and B) D11 variants of MAO‐N. Residues are colored according to their pLDDT value: > 90 is dark blue; > 80 is light blue; > 70 is green; > 60 yellow; > 50 is orange. **Supporting Fig. S2**: Mean RMSD relative to the first frame for A) repeats 1‐3 and B) repeats 4‐6. **Supporting Fig. S3**: Per‐frame distance plots measuring O_2_ travel distance with respect to binding site center of mass for A) D5 and B) D11. 1 frame = 0.2 ns (simulations were binned 100x). The darker regions indicate frames in which the O_2_ to binding site distance was below the average channel distance of 16.31 Å. Total number of frames per variant = 9000; histogram bin size = 3. **Supporting Fig. S4**: Movement of O_2_ from the center of a MAO‐N monomer binding site to the solvent surrounding the protein. O_2_ trajectories are shown as a spectrum from blue (0 ns) to red (time point at which O_2_ escaped). FADH_2_ is shown in yellow without hydrogen atoms for visual clarity. The paths taken by O_2_ include A) substrate channel 1, B) substrate channel 2, C) alternate exit 1 and D) alternate exit 2. **Supporting Table S1**: Mutated residue occurrence in the top 25 residues for channels used by O_2_ to escape.

## Funding

This study was supported by the Engineering and Physical Sciences Research Council and Royal Society of Chemistry (E20‐9653).

## Conflicts of Interest

The authors declare no conflicts of interest.

## Supporting information

Supplementary Material

## Data Availability

The data that support the findings of this study are available on request from the corresponding author. The data are not publicly available due to privacy or ethical restrictions.
