# Supplementary material for: Using MD Simulations to Understand the Impact of Directed Evolution on Oxygen Affinity in Amine Oxidases
Source: ChemistryOpen. 2026 Mar 29;15(4):e70164. doi: 10.1002/open.70164 (PMC13140974; doi:10.1002/open.70164)
Supplement: Supplementary file 1 — Supplementary Material [file OPEN-15-e70164-s001.pdf]

Using MD simulations to understand the impact of directed evolution on oxygen affinity in amine oxidases

Christopher R. Field,[a] Rowan Lindeque,[b]# Jonathan P. Dolan,[c] Julie Østerby Madsen,[b]## Sam Hay,[d] Nicholas J. Turner,[d] John M. Woodley[b]*, Sebastian C. Cosgrove[c]*

[a]: Future Biomanufacturing Research Hub, Manchester Institute of Biotechnology, Department of Chemistry, School of Natural Sciences, University of Manchester, 131 Princess Street, Manchester M1 7DN, UK.

[b]: Department of Chemical and Biochemical Engineering, Technical University of Denmark, 2800 Kgs Lyngby, Denmark.

[c]: School of Chemical and Physical Sciences and Centre for Glycoscience, Keele University, Keele, Staffordshire, ST5 5BG.

[d]: Manchester Institute of Biotechnology, Department of Chemistry, School of Natural Sciences, University of Manchester, 131 Princess Street, Manchester M1 7DN, UK.

Email: jw@kt.dtu.dk; s.cosgrove@keele.ac.uk.

# Current address: Carlsberg Research Laboratory, 1799 Copenhagen, Denmark

Current address: Novo Nordisk A/S, Denmark

Contents

General experimental 2

Practical Methods 2

Purification of MAO-N Variants 2

Michaelis-Menton kinetics for MAO-N variants 2

MAO-N Tetrahydroisoquinoline oxidation reactions 3

Computational Methods 4

$FADH_2$ and O_2 Parameterisation 4

Protein Model Preparation 4

Molecular Dynamics Simulations 4

Results 5

References 10

General experimental

Unless otherwise stated all chemicals were purchased from commercial suppliers (Acros UK, Alfa Aesar, Fisher UK, Fluorochem and Sigma Aldrich) and used as received.

Plate Assays were performed using TECAN Infinite 200 Pro M Nano.

Spectrophotometric readings were performed using a Thermo Scientific Nanodrop 1000.

SDS-PAGE was carried out using Bio-Rad mini protean 3 apparatus using Bio-Rad mini-PROTEAN TGX precast gels or freshly prepared gels. SDS-PAGE gels were stained with Coomassie blue. Buffers and media were made inhouse unless otherwise stated, using analytical grade reagents from commercial suppliers.

All common buffers and media were prepared with 18.2 MΩ water to the required volume. The pH of the solutions was adjusted using 5 M NaOH or 5 M H_3PO_4 followed by filtration using Thermo Scientific^{TM} Nalgene^{TM} Membrane and Prefilter Disks, material Nylon, pore size 0.2 µm. Media was sterilised by autoclave at 126 °C for 15 min.

Gas chromatography (GC) data was recorded using an Agilent 7820A GC-FID system. The column used was an Agilent HP-5 (30 m × 0.32 cm × 0.25 µm). The data was analysed using Agilent Open Lab software packages.

Practical Methods

Purification of MAO-N Variants

Cell-free extract (CFE) of each variant was suspended in 100 mM KPi (pH 8) to 10 mg/mL and gently rocked for 30 minutes before centrifugation at (30,000 *x g*, 25 mins, 4 °C) to remove insoluble debris. The supernatant was applied to His Pur Ni-NTA Spin column (5 mL) pre-equilibrated with 100 mM KPi (pH 8). The resin was washed with 5 column volumes of 100 mM KPi (pH 8) containing 20 mM imidazole and the protein was eluted in 5 mL fractions using 5 column volumes of 100 mM KPi (pH 8) containing 250 mM imidazole. Fractions were monitored using Bradford reagent. The protein size, expression level and purity were assessed by SDS-PAGE. Fractions containing the target protein by SDS-PAGE were combined and concentrated by centrifugal concentration using either 10 KDa MWCO Thermo Scientific^{TM} Pierce^{TM} protein concentrator PES or Amicon® Ultra-15 Centrifugal protein concentrator. The protein was desalted into 100 mM KPi (pH 8) using a Cytiva PD-10 desalting column according to the manufacturer's instructions. Protein concentration was determined by Pierce BCA Protein Assay Kits (Thermo Fisher).

Michaelis-Menton kinetics for MAO-N variants

The specific activity, kinetics constants and conversion rates of the MAO-N variants were measured using a 4-AAP-TBHBA-HRP coupled assay. A 2-fold serial dilution of Tetrahydroisoquinoline (THIQ) from 128 mM was prepared using 100 mM KPi (pH 8) containing HRP (1 mg/mL), 4-aminoantipyrine (4-AAP, 100 mg/mL) and 2,4,6-tribromo-3-hydroxybenzoic acid (TBHBA, 20 mg/mL). 90 µL of each dilution was added to a 96-well plate followed by 10 µL 1 mg/mL MAO-N (Final: 0.1 mg/mL). The production of 4-AAP/TBHBA dye (ε = 29400 L mol^{-1} cm^{-1}) was monitored at 510 nm using TECAN Infinite 200 Pro M Nano plate reader at 37 °C in triplicate. For the

kinetic characterisation of the proteins, the kinetic constants V_{max} and K_M were determined by fitting the data to the Michaelis-Menten equation using OriginPro 2019b software.
The assay was repeated in buffer (100 mM KPi (pH 8)) which had been degassed by vacuum filtration through 0.2 µm nitrocellulose membrane. For aerated buffer, compressed air was bubbled through degassed buffer for 20 minutes through an 18-gauge needle. For oxygenated buffer, pure oxygen was bubbled through degassed buffer for 20 minutes through an 18-gauge needle.

MAO-N Tetrahydroisoquinoline oxidation reactions

Reactions were carried out in a 150 mL (liquid volume) my-Control stirred tank reactor (Applikon Biotechnology B.V., Netherlands), as illustrated here.[11] An Ismatec Reglo Independent Channel Control peristaltic pump (Cole-Parmer, USA) was used to supply the reactor with an enzyme feed (0.1 g L^{-1} AmOx, 100 mM pH 7.4 potassium phosphate buffer). The reactor was sparged at 1 vvm (volume gas per volume reaction liquid per minute) with gas (an 4:1 mixture of nitrogen and oxygen to represent atmospheric air composition) and was agitated at 1000 rpm to ensure well-mixed conditions. Foam formation in the reactor was controlled by manual dropwise addition of Antifoam 204. Oxygen saturation (%) in the reactor was monitored and logged using a robust optical oxygen probe (Pyroscience AT GmbH, Germany). The probe was calibrated by saturating the reaction media, prior to initiation of the reaction by addition of the enzyme, with nitrogen to achieve 0% oxygen saturation and separately, pure oxygen to achieve 100% oxygen saturation. Percentage oxygen saturation was converted to dissolved oxygen concentration (mM) using the Henry's Law constant of oxygen in water (1.2×10^{-5} mol m^{-3} Pa^{-1}). During operation, samples (490 µL) were taken from the reactor at regular intervals, using 5 M NaOH (10 µL) to quench the reactions and then EtOAc (500 µL) to extract the organics. The samples were analysed by GC-FID: Injector: 220 °C, Detector: 250 °C, method: 80 °C, then 5 °C ramp per minute to 150 °C and hold for 2 minutes. Agilent HP-5 column, THIQ = 8.47 mins, DHIQ = 7.93 mins.

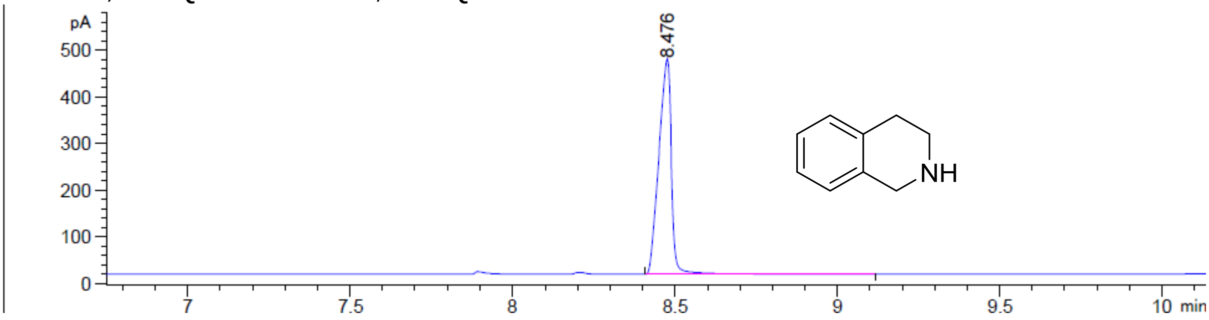

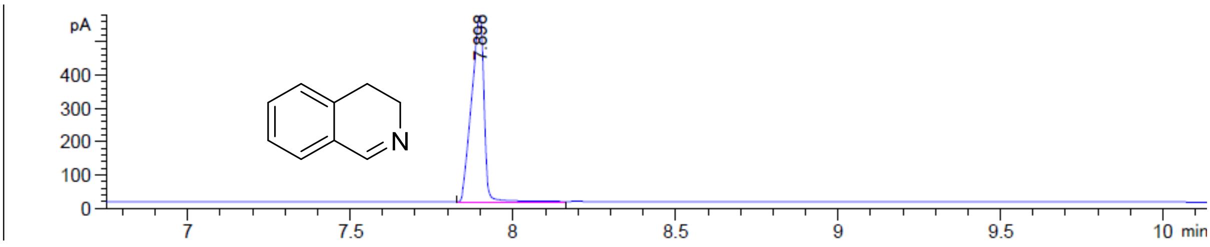

Computational Methods

$FADH_2$ and O_2 Parameterisation

Structures for $FADH_2$ and O_2 were generated using *Avogadro* v.1.99 [1], then parameterised with the *ANTECHAMBER* and *PARMCHK2* modules in *AmberTools23* [2] using *General AMBER Force Field 2* (*GAFF2*) [3]. Partial charges for $FADH_2$ were calculated using the *AM1-BCC2* charge method [4].

Protein Model Preparation

Structures for the D5 and D11 variants of MAO-N were built as dimers using crystal structures *2VVM* [5] and *3ZDN* [6], respectively. Dimeric structures were used for both variants to balance the number of binding sites simulated with the number of atoms per simulation. The missing residues in each structure (*2VVM*: chain A 32-37 and 488-495, chain B 32-39 and 487-495; *3ZDN*: chain A 32-39 and 487-495, chain B 32-39 and 486-495) were added with *ColabFold* [7], using their respective crystal structures as templates. Hydrogen atoms were added to each model using the *H++* server [8], assuming a pH of 8.0. Unlike in most MAO enzymes, the flavin is not covalently bound in MAO-N [5], as can be observed in the crystal structures.

$FADH_2$ molecules were positioned in each flavin binding site by first aligning the top-ranked structures (highest average predicted local distance difference test (pLDDT)) for both variants with their corresponding template crystal structures using the *align* command in *PyMOL* [9], then aligning the $FADH_2$ molecules with the existing FAD molecules in the crystal structures with the same command. An O_2 molecule was placed at the geometric centre of each substrate binding site (2 per simulation), as calculated using the binding site residues for each monomer (94, 213, 230, 245, 246, 430 and 466) and the isoalloxazine atoms of the proximal $FADH_2$ molecule.

Molecular Dynamics Simulations

Simulation systems were prepared with the *LEaP* package in *AmberTools23* using the Amber *ff14SB* force field [10]. Each system was equilibrated in a cuboid periodic box and solvated using the *TIP3P* water model with an internal offset distance of 10 Å. The average bounding box volumes were 1.674×10^7 ($\pm 1.132 \times 10^6$) Å^3 across D5 simulations and 1.766×10^7 ($\pm 5.984 \times 10^5$) Å^3 across D11 simulations. The systems were then neutralized and adjusted to a final salt concentration of 150 mM with explicit counterions (K^+ and Cl^-) using the *SPLIT* method [11]. Simulations were run in triplicate using the *CUDA* implementation of the *Particle Mesh Ewald Molecular Dynamics* (*PMEMD*) engine in *AMBER 20* [12]. Simulations were run for 200 ns at 310.15 K without restraints using unique starting seeds. The final frame of each replicate was used as

the starting structure for an additional 100 ns simulation under the same conditions. O_2 molecules were repositioned at the binding site centres in the starting structures for the 100 ns simulations. Root mean square deviation (RMSD) and O_2 residence times were calculated using *cpptraj* from *AmberTools*. Channel calculations were performed using *CAVER analyst 2.0* [13]. Channel starting points were set to the centre of each binding site (94, 213, 230, 245, 246, 430 and 466 for each monomer).

Results

ColabFold structures for both variants had high similarity with their crystal structures

The *ColabFold* structures for the complete D5 (Figure S1a) and D11 (Figure S1b) sequences had average pLDDT values of 97.30 and 97.29, respectively. A significant decrease in per-residue pLDDT was observed between residues 32-40 (75.92 ± 11.18) and at the C-terminus (48.79 ± 23.04) of each monomer for each variant. These regions correspond with unresolved regions in the template crystal structures. Their absence in the crystal structures and positioning on the protein surface heavily suggest these regions are highly mobile and unstructured, explaining their low confidence scores. Given that these regions occur on the opposite side of the proteins as the previously identified substrate channels [14], they were expected to have no impact of the results of the simulations. The RMSD between *2VVM* and the *ColabFold* D5 model was 0.282 Å, and between *3ZDN* and the *ColabFold* D11 model was 0.271 Å, both across 990 residues, demonstrating good agreement between the *ColabFold* and crystal structures. Any small variations between the templates and complete models were expected to have no impact on the simulations.

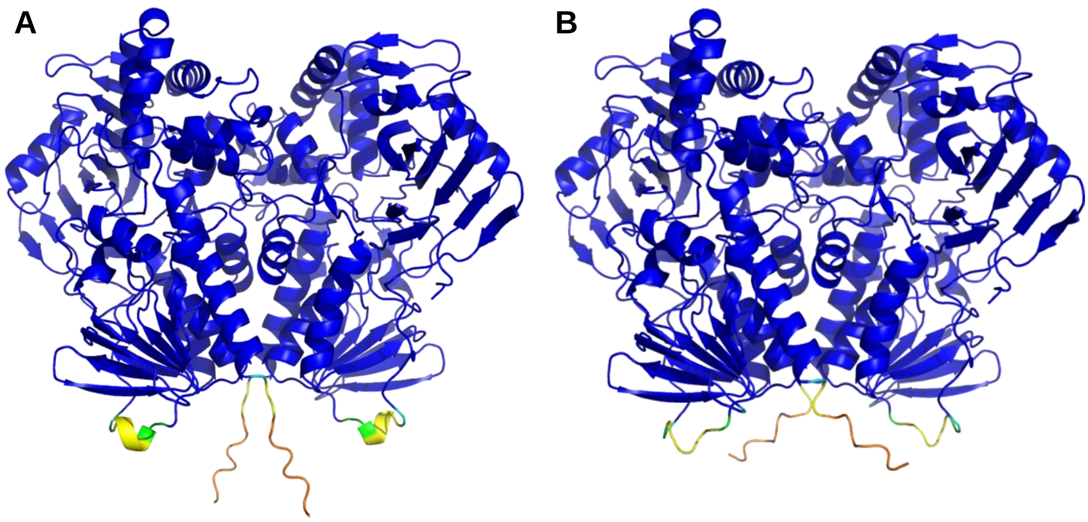

Figure S1. *ColabFold* structures of the complete sequences for the A) D5 and B) D11 variants of MAO-N. Residues are coloured according to their pLDDT value: > 90 is dark blue; > 80 is light blue; > 70 is green; > 60 yellow; > 50 is orange.

Protein and cofactor conformations remained consistent during simulations

RMSD values were calculated for the protein dimers and $FADH_2$ molecules relative to their positions at the initial timestep (Figure S2a and S2b). The mean RMSD across all 990 protein residues converged at 3.09 ± 0.14 Å (repeats 1-3) and 2.54 ± 0.25 Å (repeats 4-6) for D5, and at 3.31 ± 0.33 Å (1-3) and 2.34 ± 0.30 Å (4-6) for D11. The $FADH_2$ molecules converged at 1.17 ± 0.11 Å (1-3) and 0.83 ± 0.11 Å (4-6) for D5, and at 0.80 ± 0.11 Å (1-3) and 1.05 ± 0.15 Å (4-6) for D11. Overall, this shows no major conformational changes occurred over the course of the simulations.

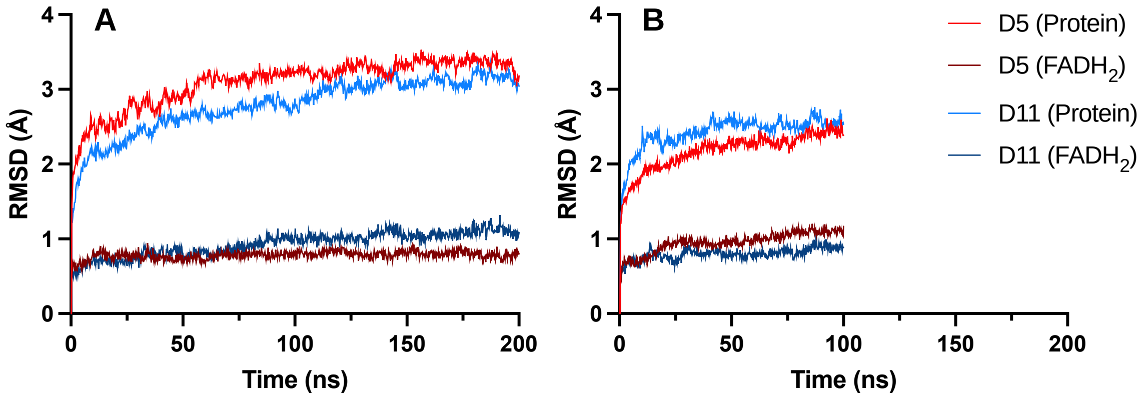

Figure S2. Mean RMSD relative to the first frame for A) repeats 1-3 and B) repeats 4-6.

O_2 residence times were lower in D11 simulations

Across 12 simulations (6 per variant, 2 binding sites per simulation), a total of 16 of a possible 24 O_2 molecules escaped from their originating MAO-N monomer into the surrounding solvent. 7 escapes occurred in D5 simulations, and 9 escapes occurred in D11 simulations. Average O_2 residence times for D11 simulation (35.51 ns) was less than half that of the D5 simulations (72.09 ns). O_2 molecules remained within the binding site or substrate channels across a larger proportion of frames in D5 simulations (Figure S3a) than in D11 simulations (Figure S3b) (65.10% and 42.79%, respectively). Overall, these data align with the experimental findings that the D11 variant has a lower affinity for O_2 than D5.

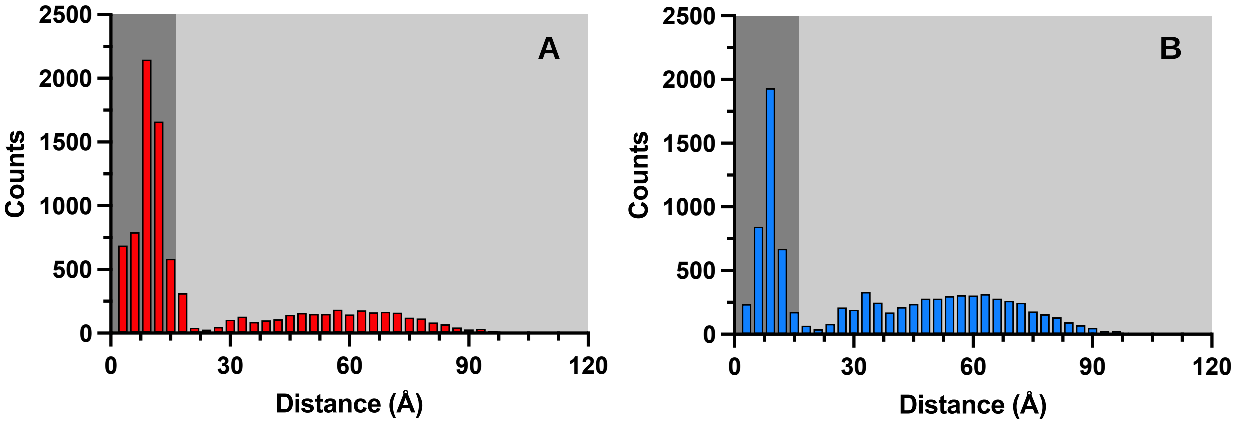

Figure S3. Per-frame distance plots measuring O_2 travel distance with respect to binding site centre of mass for A) D5 and B) D11. 1 frame = 0.2 ns (simulations were binned 100x). The darker regions indicate frames in which the O_2 to binding site distance was below the average channel distance of 16.31 Å. Total number of frames per variant = 9000; histogram bin size = 3.

O_2 primarily occupied and escaped via the primary substrate channel, using the opening present at the MAO-N dimer interface, in 10/16 escapes (5 for each variant) (Figure S4a). The secondary substrate channel, previously described by Curado-Carballada *et al.* (2019) [14], was used in the remaining 4 D11 escapes (Figure S4b). This channel was not used in any D5 simulations, possibly indicating at its contribution in the observed reduction in O_2 affinity for D11. The secondary substrate channel occurs near the β-hairpin region (residues 124-141), which remained in the closed conformation during all simulations, as their durations (100-200 ns) were too short to observe the μs-scale transition between the closed and open states. In the remaining 2 D5 simulations, O_2 escaped via the outer face of the dimer through channels termed alternate exit 1 (Figure S4c) and alternate exit 2 (Figure S4d).

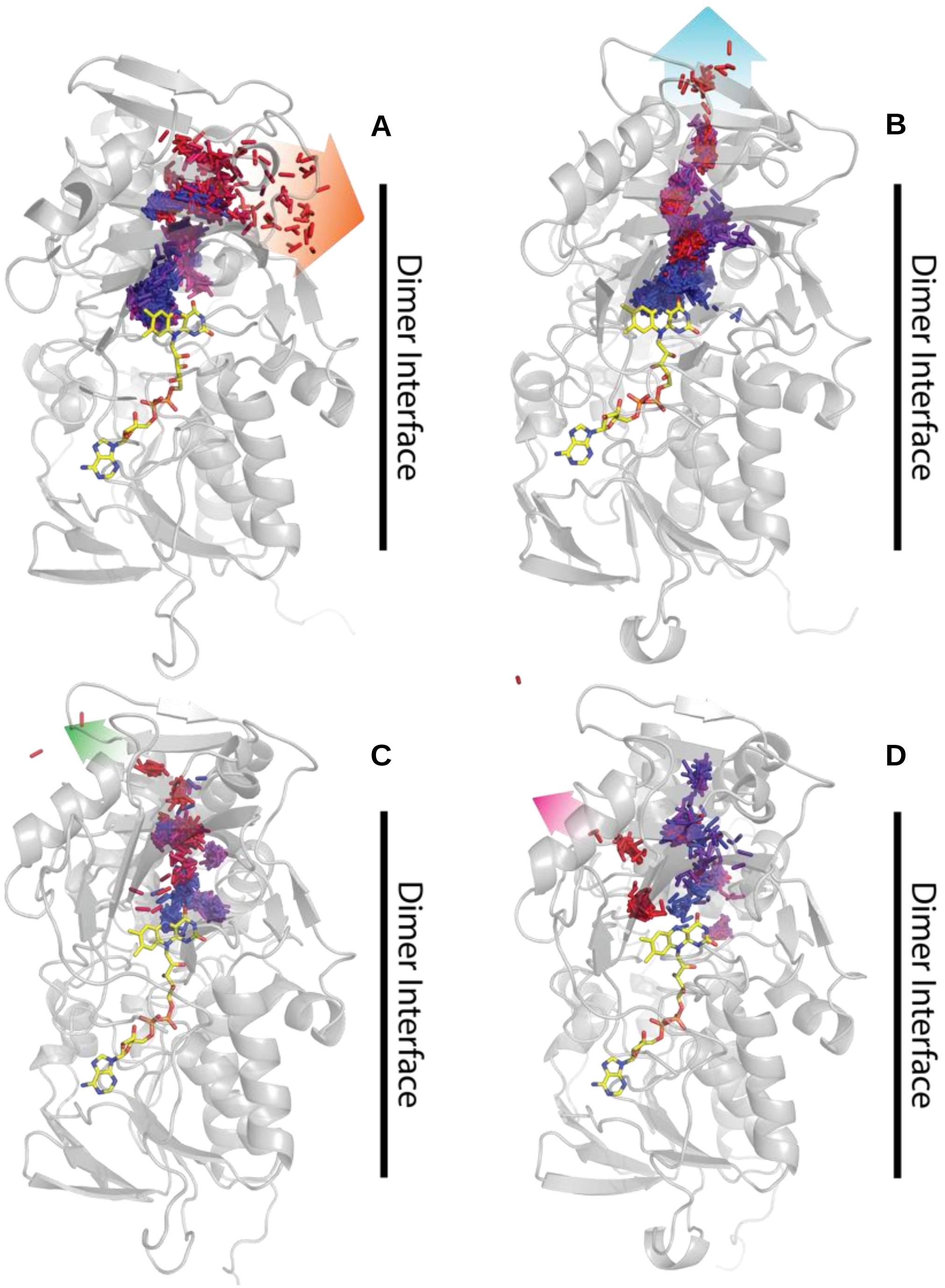

Figure S4. Movement of O_2 from the centre of a MAO-N monomer binding site to the solvent surrounding the protein. O_2 trajectories are shown as a spectrum from blue (0 ns) to red (time point at which O_2 escaped). $FADH_2$ is shown in yellow without hydrogen atoms for visual clarity. The paths taken by O_2 include A) substrate channel 1, B) substrate channel 2, C) alternate exit 1 and D) alternate exit 2.

Substrate channel mutations occurred most along O_2 escape paths

Table S1 shows the occurrence of residues mutated from the wild type among the top 25 residues present in channels used by O_2 to escape the protein. Substrate channel mutations I246M/T, F210L and L213T were the most commonly occurring O_2 escape channel residues. Mutations near the β-hairpin region were much less frequently a part of these channels, further implying the mutations present in the substrate channels of the D11 variant had the most impact on O_2 retention.

Table S1. Mutated residue occurrence in the top 25 residues for channels used by O_2 to escape.

Residue	Location	Mutation (D5)	Mutation (D11)	D5 channel (n=7)	D11 channel (n=9)	Combined (n=16)
**246**	Substrate channel	**I246M**	**I246T**	100.00%	100.00%	100.00%
**210**	Substrate channel	F210	**F210L**	85.71%	100.00%	93.75%
**213**	Substrate channel	L213	**L213T**	85.71%	100.00%	93.75%
**242**	Substrate channel	M242	**M242Q**	28.57%	100.00%	68.75%
**430**	Binding site	W430	**W430G**	100.00%	0.00%	43.75%
**336**	Adjacent to binding site	**N336S**	**N336S**	42.86%	11.11%	25.00%
**384**	Close to β-hairpin	**T384N**	**T384N**	42.86%	11.11%	25.00%
**385**	Close to β-hairpin	**D385S**	**D385S**	28.57%	0.00%	12.50%
**348**	Close to β-hairpin	**M348K**	**M348K**	0.00%	0.00%	0.00%

References

1. Hanwell, M.D., et al., *Avogadro: an advanced semantic chemical editor, visualization, and analysis platform.* J Cheminform, 2012. **4**(1): p. 17.
2. Case, D.A., et al., *AmberTools.* J Chem Inf Model, 2023. **63**(20): p. 6183-6191.
3. Wang, J., et al., *Development and testing of a general amber force field.* J Comput Chem, 2004. **25**(9): p. 1157-74.
4. Jakalian, A., D.B. Jack, and C.I. Bayly, *Fast, efficient generation of high-quality atomic charges. AM1-BCC model: II. Parameterization and validation.* J Comput Chem, 2002. **23**(16): p. 1623-41.
5. Atkin, K.E., et al., *The structure of monoamine oxidase from Aspergillus niger provides a molecular context for improvements in activity obtained by directed evolution.* J Mol Biol, 2008. **384**(5): p. 1218-31.
6. Ghislieri, D., et al., *Engineering an enantioselective amine oxidase for the synthesis of pharmaceutical building blocks and alkaloid natural products.* J Am Chem Soc, 2013. **135**(29): p. 10863-9.
7. Mirdita, M., et al., *ColabFold: making protein folding accessible to all.* Nat Methods, 2022. **19**(6): p. 679-682.
8. Gordon, J.C., et al., *H++: a server for estimating pKas and adding missing hydrogens to macromolecules.* Nucleic Acids Res, 2005. **33**(Web Server issue): p. W368-71.
9. *PyMOL*. 2023, Schrödinger, LLC. p. The PyMOL Molecular Graphics System.
10. Maier, J.A., et al., *ff14SB: Improving the Accuracy of Protein Side Chain and Backbone Parameters from ff99SB.* J Chem Theory Comput, 2015. **11**(8): p. 3696-713.
11. Machado, M.R. and S. Pantano, *Split the Charge Difference in Two! A Rule of Thumb for Adding Proper Amounts of Ions in MD Simulations.* J Chem Theory Comput, 2020. **16**(3): p. 1367-1372.
12. Case, D.A., et al., *Amber 2020*. 2020: University of California, San Francisco.
13. Jurcik, A., et al., *CAVER Analyst 2.0: analysis and visualization of channels and tunnels in protein structures and molecular dynamics trajectories.* Bioinformatics, 2018. **34**(20): p. 3586-3588.
14. Curado-Carballada, C., et al., *Hidden Conformations in Aspergillus niger Monoamine Oxidase are Key for Catalytic Efficiency.* Angew Chem Int Ed Engl, 2019. **58**(10): p. 3097-3101.
